# The need for supportive policies: connecting individual placement and support to the domains of employment and mental health care

**DOI:** 10.3389/phrs.2026.1608802

**Published:** 2026-10-15

**Authors:** Jaap van Weeghel, Angelo Fioritti, Dorothea Jaeckel, Gary Johnston, Greta Mazzetti, Minna Ahola, Ulrika Bejerholm, Cris Bergmans, Frédéric Debroas, Lars de Winter, Soffia Erla Einarsdottir, Dina Guglielmi, Hlynur Jónasson, Anna Kárníková, Débora Koatz, Julia Kropeit, Anne Lise Lunder Arvesen, Denise Manchisi, Jan Markerink, Kine Nan Lium, Noora Sipilä, Una Twomey, Chantal Van Audenhove

**Affiliations:** 1 Tranzo, Tilburg School of Social and Behavioral Sciences, Tilburg University, Tilburg, Netherlands; 2 Ypsilon Association, Bologna, Italy; 3 Vivantes Netzwerk fur Gesundheit GmbH, Berlin, Germany; 4 Social Finance, London, United Kingdom; 5 Department of Education Studies, University of Bologna, Bologna, Italy; 6 Finnish Institute for Health and Welfare, Helsinki, Finland; 7 Department of Psychiatry, Skane University Hospital, Lund, Sweden; 8 Divosa Association, Utrecht, Netherlands; 9 Working First Association, Marseille, France; 10 Kenniscentrum Phrenos, Utrecht, Netherlands; 11 Landspitali Psychiatric, Reykjavik, Iceland; 12 BONA ops, Prague, Czechia; 13 Institut Avedis Donabedian Universitat Autonoma de Barcelona, Barcelona, Spain; 14 Norwegian Labour and Welfare Administration, Oslo, Norway; 15 Ipsilon Association, Bologna, Italy; 16 Finnish Institute for Health and Welfare, Helsinki, Finland; 17 HSE National Mental Health, Dublin, Ireland; 18 LUCAS Center for Care Research & Consultancy, KU Leuven, Leuven, Belgium

**Keywords:** employment, individual placement and support (IPS), mental illness, occupational health, social recovery

## Abstract

**Background:**

Individual placement and support (IPS) is an effective vocational rehabilitation programme for people with mental illness. This article analyses national policies that directly promote IPS and broader employment-related policies on mental health in 13 European countries.

**Analysis:**

IPS alone cannot address the broader workplace and policy conditions that influence sustainable employment. Therefore, creating mentally healthy working conditions also requires action by employers, healthcare systems, and policymakers.

**Policy options:**

Recurring barriers and facilitators were identified across countries, alongside variation in governance and implementation contexts. IPS services often operate in isolation, with limited access to consistent training, data, and cross-sector coordination. A national infrastructure for IPS is therefore needed to offer implementation support, intersectoral collaboration should be strengthened and employers need to be supported to advance equity, diversity and inclusion. IPS practice will benefit from policies addressing poor mental health conditions in the workplace, and policies endorsing a recovery-oriented mental healthcare system.

**Conclusion:**

IPS can build a solid bridge between mental healthcare and employment when integrated into recovery-oriented services and supported by policies that promote inclusive, mentally healthy workplaces.

## Background

Employment is viewed as an indicator of competence, self-reliance, health, and social connectedness, and significantly influences our mental and emotional wellbeing [1, 2]. For people with (severe) mental illness, having a job can be particularly valuable: it can help them better manage symptoms, increase motivation, and reduce public and self-stigma [3, 4]. However, their labor force participation has remained limited over the years: 15%–25% of people with severe mental illness have regular employment [5].

The relationship between employment and mental health is complex. On the one hand, employment, under favourable conditions, leads to better (mental) health. It is therefore understandable that clients under mental healthcare wish to have a regular job. On the other hand, some degree of (mental) health is required to remain employed. In European countries employment places high demands on workers’ qualifications, self-reliance, and mental health. Mental ill-health is associated with high levels of sickness absence and premature labour-market exit [6, 7].

To overcome potential barriers individuals with mental illness need access to effective programmes offering multi-faceted support to get and keep the jobs they want. Individual placement and support (IPS), originally developed for people with severe mental illness, is such a programme. Central to IPS is the principle that participants work in jobs they want with tailored support. Training in advance is not required: clients start working in the chosen position, then trained in the necessary work skills (‘first-place-then-train’). Employment specialists offer tailored work support to clients and employers. In IPS employment support and mental healthcare are integrated [8]. These two forms of assistance are mutually reinforcing. IPS adds a crucial component to mental healthcare by supporting clients’ social recovery through work. In turn, mental health treatment underpins clinical recovery and helps working clients self-manage their condition in the workplace.

Many studies have provided evidence of the effectiveness and cost-effectiveness of IPS [9, 10]. However, it has been demonstrated that particularly strong employment protection legislation reduces the relative efficacy of IPS [11, 12]. Although the evidence is stronger for people with severe mental illnesses, IPS is also effective for people with common mental disorders [9, 13]. The latter may also benefit from interventions such as employee assistance programmes and psychotherapeutic care in the workplace [14]. Although IPS is growing in some countries, only a small fraction of the potential target group is reached. Despite international recommendations and frameworks promoting integrated approaches to mental health and employment [15, 16], national policies to maximize the availability of IPS are often not in place.

This article presents a consensus-based comparative policy synthesis regarding the implementation and scaling of IPS in 13 European countries. Research on this topic is still scarce, and often concerns countries outside Europe [17, 18]. Country representatives completed a structured template covering the status and governance of IPS policy, implementation barriers, policy priorities, and alignment with workplace mental-health and recovery-oriented care. Responses were compared across countries, grouped into recurring categories, and circulated for country-level verification and consensus. All authors are active in the European IPS learning network [19]. They are involved in IPS research, implementation, or policy development in their countries, representing both early and recent adopters of IPS. They originate from ten member states of the European Union (EU): Belgium, Czech Republic, Finland, France, Germany, Ireland, Italy, Netherlands, Spain and Sweden. Three countries (Iceland, Norway, UK) have extensive connections with the EU. This article is addressed to European and national policymakers across (mental) health, employment, and social welfare domains.

## Analyses

IPS is a pragmatic, easily understood approach, using face valid employment outcomes, which makes it appealing to policymakers and the public. However, more is needed to disseminate IPS. Key facilitating factors include supporting government policies and institutional regulations, sustainable funding, leadership and local champions, service integration, technical assistance centres, IPS learning communities, destigmatisation efforts, and replication of outcome studies [20–22]. To establish these facilitators strong coalitions and shared ownership of IPS are needed. Ideally, key stakeholders from mental health, employment and social welfare services, as well as representatives of the target group and their families, participate in this coalition.

Developing policies to promote IPS is necessary but not sufficient to achieve sustainable labor participation. The interplay between mental ill-health and employment has emerged as a major global concern in recent years. Two main perspectives are relevant. The medical-pedagogical approach places responsibility for addressing work-related conditions on individual employees. They must learn to manage their health condition and address gaps in experience, resilience, self-confidence and skills. In the sociological perspective, the cause lies in societal institutions, which are not inclusive and exclude people with disabilities [23].

Pursuing ‘decent work’ is a valid starting point to promote mental health in the workplace. Decent work, defined as “productive work for people in conditions of freedom, equity, security and human dignity” [23], is included in the European Social Charter. Its elements include job creation (no one should be barred from their desired work due to lack of employment opportunities), rights at work (the right to minimum wage, just and favorable conditions, days off, 8-h days, non-discrimination and living wages for them and their families), social protection (workers should have safe working conditions, adequate free time and rest, access to healthcare, pension, and parental leave), and social dialogue (workers should be able to exercise workplace democracy and negotiate their workplace conditions as well as national and international labor and development policies). Decent work is the eighth objective of the United Nations Sustainable Development Goals. The fourth objective highlights the importance of lifelong learning. Combining these objectives is crucial for further developing IPS. Many young clients do not have the required starting qualifications and first need to complete a study or training course.

Employment may promote or undermine mental health. Concerns about relapse, loss of benefits, and stigma may contribute to ambivalence about returning to work [24]. The World Health Organization (WHO) and the International Labor Organization (ILO) stated that workers with mental illness need support to fully participate in work through reasonable accommodations, return-to-work programmes and supported employment [25]. Poor working conditions can, however, negatively affect employee retention and mental health. To prevent work-related mental health problems, psychosocial risk management is needed, including interventions to reshape working conditions, cultures and relationships. Furthermore, mental health at work should be actively promoted through interventions that improve mental health literacy, strengthen skills to act on mental health conditions at work, and empower workers to seek support [26]. This requires an environment in which actions are taken to improve mental health at work through leadership, investment, rights, integration, evidence and compliance [27]. In summary, an integrated approach is needed that addresses workers’ mental health and employment challenges concurrently [16].

In 2024, the Belgian Presidency of the European Union took the initiative to advance these ambitions [28]. The aim was to formulate a European directive that supports member states’ efforts in two directions: to prevent the onset of mental ill-health linked to working conditions (primary prevention); and to create an inclusive labour market that supports groups at high risk of exclusion (secondary and tertiary prevention). Priority was to identify policies promoting sustainable employment and personal growth adapted to the unique needs of each individual.

To contribute to these ambitions, recovery-oriented mental healthcare is needed, focusing on hope, agency, wellbeing, and social inclusion rather than just symptom reduction. A promising approach is outlined in the position paper of the network of European Community-based Mental Health Service Providers (EUCOMS) “Recovery for all in the community” [29]. This approach is based on human rights, uses a public health perspective, offers recovery-oriented care and effective interventions, builds community care networks, and utilizes peer expertise on all levels. IPS emerged from the professional culture of mental healthcare that seeks to embody these key elements. In general, current consolidated principles of mental healthcare include the adoption of person centred care and recovery plans based on shared decision-making. IPS aligns with this by empowering people to find their own way in their employment career.

## Policy options

To what extent are the aforementioned approaches accepted policy options in the participating countries? Currently, national policies on IPS exist in six countries. In some countries (Finland, Ireland, UK) the Ministry of Health is the primary driver of such policy. In the Netherlands the Ministry of Employment and Social Affairs took the lead, while in other countries (Iceland, Norway) this is a combined effort of the Ministries of Health and of Employment.

Establishing a national policy targeting IPS is important, but will not immediately remove all obstacles. Implementation barriers (a maximum of two could be reported from each country) are more similar than different across countries. Regardless of whether IPS is included in national policies or is mainly developed through regional initiatives (Czech Republic, Italy, Spain, Germany, Sweden), insufficient cooperation between mental health, employment and social welfare services is the most common obstacle (9 out of 13 countries). The absence of stable funding mechanisms (7/13), persisting “train-then-place” cultures (6/13) and limited implementation capacity or access (5/13) were also frequently identified as major barriers. These findings point to a policy–practice gap. Formal recognition of IPS does not automatically ensure consistent implementation.

Against this background, the country reports suggest a recurring, though by no means universal, pattern in the structural embedding of IPS: local initiatives and professional advocacy may precede formal recognition, followed in some countries by greater attention to sustainable funding, quality assurance, workforce development, and intersectoral governance. Countries with more established IPS infrastructures increasingly address issues beyond programme expansion, such as job retention and job quality (UK), or national fidelity monitoring and advanced training for IPS workers (Iceland, Netherlands, Norway, UK).

Decentralised governance creates both opportunities and challenges. In Italy, Spain and Sweden regional autonomy has facilitated bottom-up implementation, but also generated territorial inequalities and fragmented access to IPS. Several contributors suggested establishing national frameworks that define core IPS standards while allowing regional adaptation according to local contexts.

Contributors emphasised that IPS alone cannot address the broader workplace and system-level conditions required for decent and sustainable employment. Addressing these issues requires a more holistic approach. Sustainable implementation and scaling of IPS are facilitated when it is embedded within recovery-oriented mental health services and supported by coherent employment, disability and social policies. Several contributors noted national developments aligning with the WHO/ILO and European mental health at work agendas. However, these developments were frequently not directly based on these initiatives, and their implementation in national policies varied significantly. For example, in France conditions resulting from prolonged exposure to psychosocial risks at work are recognised as occupational diseases. In Germany, employers are required to assess psychological strain as part of occupational risk assessment. Norway has introduced employers’ responsibility to identify and manage risk factors such as excessive workload, time pressure, unclear expectations, lack of support, and harassment.

Despite these initiatives, most countries lack a unified strategy or a cross-government framework on mental health at work. It is important to maintain a clear conceptual distinction between IPS and broader workplace mental health policies. IPS cannot by itself create decent work. However, IPS can help individuals with mental illness access and sustain quality employment by offering individualised support to both these workers and their employers, including workplace accommodations and problem-solving. The integration of employment support and mental healthcare enables workers to manage their illness while remaining employed.

Contributors agreed on a multifaceted strategy to promote the dissemination and quality improvement in IPS. First, several contributors recommended formal recognition of IPS as a preferred evidence-based intervention. A policy framework can then be developed for a sustainable IPS infrastructure (suggestion from Germany). IPS should be an obligatory standard in the accreditation system for mental health services (suggestion from Italy). Experiences from Norway suggest that once stable funding mechanisms for IPS are in place, other implementation barriers become increasingly important: clear governance structures and shared accountability between employment and health services to support integrated IPS delivery; national solutions for information sharing and collaboration, including clear regulations regarding confidentiality, consent, and access to relevant information across sectors.

Second, the implementation and quality assurance of IPS should be organised at the national level, offering implementation support, fidelity monitoring, and a research programme. This strengthens the sustainability of IPS services and reduces regional inequalities (suggestion from Sweden and UK). It would also facilitate investments in the IPS workforce by offering national training pathways, accreditation and career progression routes (suggestion from UK). Third, intersectoral collaboration should be strengthened. This requires formalising intersectoral governance mechanisms connecting mental health services, employment services, social services, and employers (recommendation from Czech Republic, Finland and Spain). Also needed are shared outcome measures across sectors, encouraging services to work towards common goals regarding recovery, education, and employment (recommended from Norway). Fourth, IPS should be accessible to all individuals who need it. To this end, employment outcomes need to be incorporated into the mental health system’s accountability (suggestion from Spain). And IPS should be positioned as a core intervention in national responses to rising inactivity due to mental ill health (proposed from Italy and UK). Last, it is essential to engage and support employers. Both engagement and incentives are needed to promote equity, diversity and inclusion (suggestion from Czech Republic, Finland, France, Iceland, and Netherlands). More is expected of employers addressing workload, job control, and workplace culture alongside individual level interventions (suggestion from UK).

## Conclusion

Our findings from 13 European countries reveal consistent patterns of barriers and facilitators in disseminating IPS across different welfare systems and governance arrangements. IPS should be granted the status of preferred intervention, and a national infrastructure is needed to offer implementation support. Intersectoral collaboration should be strengthened, and employers need support to advance equity, diversity and inclusion. Findings also confirm our central argument that future developments of IPS depend not only on expanding service availability, but also on creating coherent policies capable of integrating evidence-based employment support within broader systems of mental healthcare and labour market inclusion. The challenge is to translate policy commitments into governance arrangements, sustainable financing, workforce capacity, and mechanisms for implementation support and accountability.

We are aware that this expert-informed comparison has its limitations. The involvement of all contributors in the European IPS learning network provides substantial country-specific implementation knowledge, but may also introduce an IPS-oriented perspective in the selection and interpretation of policy priorities. Furthermore, healthy workplaces and recovery-oriented mental healthcare are not the only contexts needed to support IPS and its clients. Other issues should also be addressed, in particular policies counteracting stigma (30): not only interpersonal stigma (negative attitudes of employers and coworkers towards people with mental illness), but also organisational stigma (biased hiring and promotion practices) and structural stigma (disadvantages ingrained in policies, institutions, and socio-cultural norms).

IPS is certainly not a panacea for the vocational integration of people with mental illness. But by offering an ‘active labour market strategy’ to people with mental illness, and employment being a ‘critical health intervention’, IPS can act as a catalyst for closer collaboration between health, employment and social policy sectors.
